# Community Vaccinators in the Workplace

**DOI:** 10.3201/eid1706.101763

**Published:** 2011-06

**Authors:** Jeffrey R. Harris, Diane Martin, Patricia Lichiello, Faruque Ahmed, Carol Friedman, Barbara Williams

**Affiliations:** Author affiliations: University of Washington School of Public Health, Seattle, Washington, USA (J.R. Harris, D. Martin, P. Lichiello, B. Williams);; Centers for Disease Control and Prevention, Atlanta, Georgia, USA (F. Ahmed, C. Friedman)

**Keywords:** Vaccination, immunization, human influenza, workplace, disease outbreaks, whooping cough, diphtheria, tetanus, vaccine, letter

**To the Editor:** Adult vaccination rates are low (*1*), and workplaces are a useful location for increasing vaccination (*2*). In 2008, only 41% of US workers 50–64 years of age reported vaccination against influenza virus (*3*). Workplace vaccination is common and increases with employer size (*4*). Among adults, the workplace is the most common site for influenza vaccination for persons 18–49 years of age and second most common for persons 50–64 years (*2*). Offering vaccination in the workplace increases vaccination coverage (*5*).

Consistent with guidelines and economic incentives, employers have focused workplace vaccination on seasonal influenza (*4*), but the workplace has also been a key site for vaccination against influenza A pandemic (H1N1) 2009 and could be a site for other adult vaccinations. The most recent guidelines from the Advisory Committee on Immunization Practices recommend annual influenza vaccination of all adults (*6*). In most years, the seasonal influenza vaccine and predominant circulating viruses are well matched, and employers have an economic incentive to decrease worker absenteeism by increasing influenza vaccination (*7*). The workplace is also potentially a site for delivery of herpes zoster, pneumococcal, and tetanus-diphtheria-pertussis vaccines (*6*).

Our experience with employers suggests that most contract with external organizations (i.e., community vaccinators) to provide workplace vaccination, but we found little or no information about these organizations in the literature. Therefore, we interviewed community vaccinators about their 2009 experience with workplace vaccination against seasonal influenza virus and pandemic (H1N1) 2009 virus, their business practices, barriers encountered, and delivery of other adult vaccines.

We selected a diverse study population of community vaccinators. We combined the 10 US Department of Health and Human Services regions to create 5 study regions. Beginning with a list of vaccinators provided by the Centers for Disease Control and Prevention (Atlanta, GA, USA) and searching with Google for “on-site vaccinators,” we identified 17 national and 28 local vaccinators (full list available from the authors). We selected at least 1 national and 1 local vaccinator from each region and then purposively sampled them to increase geographic and organizational diversity. Our sample comprised 5 national vaccinators, 7 local vaccinators serving urban and rural workplaces, a mobile-clinic vaccinator, a visiting nurses association, and an occupational health specialist.

The qualitative study (*8*) used a structured telephone interview with community vaccinators’ lead personnel responsible for worker vaccination. Our theoretical approach was content analysis. After 2 pilot interviews, 2 interviewers completed 10 additional interviews. Because the questions in the pilot and final interviews were similar, we analyzed both groups together and report here on all 12 interviews. We designed the interviews to last <20 minutes and conducted them during March and April 2010. One interviewer used Atlas.ti software (Atlas.ti Software Development, Berlin, Germany) to code the interviews, with review and concurrence from the second interviewer. The Human Subjects Division of the University of Washington approved this study as exempt from review.

Challenges reported for the 2009 influenza vaccination season included the need for workers to receive 2 vaccines (seasonal and pandemic [H1N1] 2009) and a mismatch between vaccine demand and supply, resulting in delayed or lost business (9/12 respondents). Some vaccinators found the season more challenging than in prior seasons (4/5 national; 2/7 local), yet most reported having added clients (4/5 national; 4/7 local).

Vaccinators’ reported business practices include vaccinating at sites in addition to workplaces, for example, churches and faith-based settings (9 vaccinators), schools (9), and community centers (8). Most (9) reported vaccinating on multiple work shifts and at multiple worksites. Ten vaccinators also reported they can help employers publicize workplace vaccination events. Most did not report patient-level vaccination information to health plans (10 vaccinators), primary-care providers (9), or registries (8). Many directly bill Medicare (8) and private insurers (7) if asked.

Additional findings related to barriers and delivery of other vaccines. Commonly reported barriers to increasing workplace vaccination rates were worker reluctance (voiced as “I’m too busy,” “I don’t need it,” or “It gives me the flu”) (10 vaccinators); worker out-of-pocket costs (9); and low worker awareness of workplace vaccination events (5). Other vaccines offered by these workplace vaccinators included the following: tetanus-diphtheria-pertussis (10 vaccinators), pneumococcal (10), hepatitis A and B (7), and herpes zoster (4).

This qualitative study, although small and not necessarily representative, found remarkable consistency across community vaccinators. Vaccinators were challenged by the pandemic (H1N1) 2009 vaccination season, but the season also provided new clients. Most reported vaccinating at diverse sites in addition to workplaces, and most already vaccinated against diseases other than influenza. Vaccinators consistently identified workers’ reluctance and out-of-pocket costs, and poor publicizing of workplace vaccination events as remediable barriers to vaccination. Tackling of these barriers is supported by the literature (*9**,**10*) and the Guide to Community Preventive Services (*5*).
